# Ion Release from Endodontic and Restorative Dental Materials: Effects of pH and Time

**DOI:** 10.3390/ma18091901

**Published:** 2025-04-22

**Authors:** Zbigniew Raszewski, Katarzyna Chojnacka, Małgorzata Ponto-Wolska, Marcin Mikulewicz

**Affiliations:** 1 Everall7, Augustówka 14, 02-981 Warsaw, Poland; zbigniew.raszewski@everall7.pl; 2 Department of Advanced Material Technologies, Faculty of Chemistry, Wroclaw University of Science and Technology, Smoluchowskiego 25, 50-372 Wroclaw, Poland; katarzyna.chojnacka@pwr.edu.pl; 3 Department of Dental Propaedeutics and Prophylaxis, Warsaw Medical University, Nowogrodzka 59, 02-006 Warsaw, Poland; malgorzata.ponto-wolska@wum.edu.pl; 4 Department of Dentofacial Orthopedics and Orthodontics, Division of Facial Abnormalities, Medical University of Wroclaw, Krakowska 26, 50-425 Wroclaw, Poland

**Keywords:** glass-ionomer sealer, phosphate sealer, zinc oxide-eugenol sealer, ion release, mechanical properties

## Abstract

Background: Root canal sealers remain in long-term contact with dental tissues, raising concerns about their potential adverse effects. Methods: This study evaluates the physicochemical properties and ion-release profiles of three dental materials: zinc oxide/eugenol-based sealer, zinc phosphate cement (luting agent), and glass-ionomer cement (restorative material) under acidic (pH 4) and neutral (pH 7) conditions over 24 h and 30 days to determine their behavior and bioactivity in vitro. The materials were evaluated for their setting time, consistency, film thickness, solubility, and ion release using atomic emission spectrometry. The influence of pH and exposure time on ion release was analyzed using multiple regression analysis. Results: All tested materials met the ISO standards for their respective categories. The zinc oxide/eugenol and zinc phosphate cements released increased levels of zinc in acidic environments (pH 4), suggesting potential antimicrobial properties. The glass-ionomer cement exhibited higher silicon and strontium release under a neutral pH (pH 7), indicating potential remineralization effects. Silver from the zinc oxide/eugenol material was below the detection limit of the applied method, suggesting minimal ion release under the tested conditions. Maximum zinc release from root canal sealer occurred after 30 days at pH 4 (1.39 ± 0.26 mg), while the highest silicon release from glass-ionomer cement was observed at pH 7 after 30 days (1.03 ± 0.21 mg). Conclusions: Zinc oxide/eugenol materials exhibited increased zinc release under acidic conditions. In contrast, the restorative and luting materials demonstrated distinct ion-release patterns, aligning with their respective intended applications rather than endodontic purposes.

## 1. Introduction

Endodontic treatment is commonly required due to advanced caries, which initially affects the dentin and subsequently spreads to the pulp and root canals. Removal of infected pulp from the chamber and canals using instruments and disinfection occurs using sodium hypochlorite solution when eliminating bacteria and remaining pulp. During the next step, the canals are filled with biocompatible gutta-percha and sealers to prevent reinfection. This procedure is widely performed, with approximately 15 million root canal treatments conducted annually in the United States [1]. One of the main challenges in endodontic treatment is achieving a successful seal of the root canal. Various methods, such as hot and cold lateral gutta-percha condensation, are commonly used to fill root canals [1]. Cold lateral condensation is particularly popular because of its accessibility and lack of need for specialized equipment. Clinically, this technique involves placing gutta-percha points in the canal, with a sealant applied around the cone to fill microspaces [2].

Root sealers are available for both temporary (phosphate, glass ionomer) and long-term use (ceramic, zinc oxide-eugenol) [3,4]. Phosphate-based cements have been used for more than a century. They are valued for their rapid set time and initial low pH, which provide an antimicrobial environment. However, these cements can cause temporary irritation that stabilizes after a few days, but they may also shrink and crack over time, compromising the seal and increasing the risk of bacterial reinfection [5]. Glass ionomer-based sealers (GiCs) gradually dissolute over time, but have the advantage of chemically bonding to enamel and dentin through polyacrylic acid, enhancing adhesion and reducing microleakage [5,6].

ZnO/eugenol-based sealers exhibit favorable setting times, high stability, and intrinsic bactericidal properties attributed to zinc oxide and eugenol [7]. Eugenol disrupts bacterial cell membranes, limiting microbial growth. Although it can show cytotoxicity in vitro, dentin mitigates this effect by blocking its migration through dentin tubules. Adding metallic silver enhances antimicrobial efficacy but may cause tooth discoloration due to ion migration [8,9,10]. Some formulations contain natural resins, such as colophony and Canada balsam, which may trigger allergic reactions [11].

Bioceramic-based sealers are highly regarded for their biocompatibility and ability to promote tissue regeneration. The materials contain both bioactive and bioinert components, including alumina, zirconium oxide, and bioactive glass. Bioactive components, such as glass ceramics and hydroxyapatite, interact with tissues to promote mineral growth. The high calcium ion content creates an alkaline pH during setting, conducive to tissue repair, though extended setting times can be a drawback [12].

Despite advancements, the choice of the sealer depends on the clinical requirements and practitioner expertise. As noted by Kumar and Shruthi, no single material possesses all the ideal properties [3]. Modern dentistry emphasizes the safety and bioactivity of materials, focusing on their potential to release ions that affect tissues and fluids [9].

This study compares the physicochemical properties and ion release of phosphate-based, glass-ionomer-based, and ZnO/eugenol-based sealers, examining their behavior under varying pH conditions over time. The null hypothesis is that ion release does not significantly vary by composition or pH. It is expected that ZnO/eugenol-based sealers will release more zinc in acidic conditions, enhancing antimicrobial properties, while glass-ionomer-based sealers will release fluoride, silicon, and strontium at a neutral pH, promoting remineralization. This study aims to identify properties that optimize infection control and tissue regeneration in endodontic treatment.

This study provides a long-term quantitative analysis of the ion release of three dental materials under controlled pH conditions. Unlike previous research focused on short-term solubility or mechanical properties, this work examines ion elution kinetics and inter-element interactions over an extended period. The findings improve our understanding of pH-dependent degradation, antimicrobial effects, and remineralization potential, aiding material selection for clinical applications. This study addresses the gap in long-term ion-release analysis of endodontic and non-endodontic materials under varying conditions. Most of the research focuses on short-term solubility and cytotoxicity. This study provides a quantitative assessment of ion elution over time, considering pH-dependent stability, antimicrobial potential, and remineralization effects. The results contribute to better material selection in clinical practice. The thesis put forward at the beginning of the research is that all cemeteries will meet the normative requirements and that the amount of ions released over time is independent of the pH of the environment.

## 2. Materials and Methods

**Figure 1:**
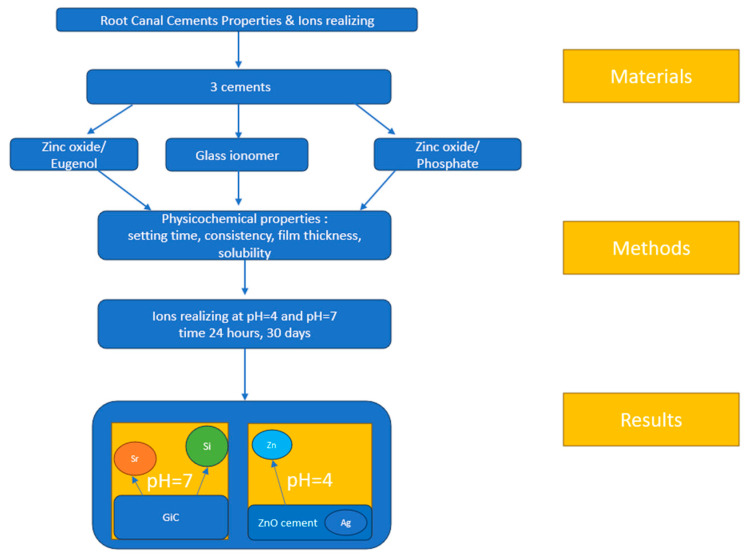
Graphical abstract: sample preparation, ISO testing requirements, and ion-release testing process for root canal sealers.

Three commercially available materials were tested: Pulp Canal Sealer (Kerr, Scafati, Italy), Kavitan (SpofaDental, Jicin, Czech Republic), and Adhesor (SpofaDental, Jicin, Czech Republic). The composition of these materials is shown in Table 1. Samples were prepared according to the manufacturer’s instructions and stored at 23 °C and 50% humidity for 72 h to ensure consistent curing conditions before testing. All sealers were two-component materials that required the mixing of powder and liquid according to the manufacturer’s specifications. The mixing was carried out in a controlled environment (23 °C, 50% humidity) to minimize the variability in the setting reaction. The mixing ratios, shown in Table 1, were determined by weighing the components with a precision balance of 0.01 g (Precisa, Dietikon, Switzerland). The liquid was dispensed dropwise, while the powder was measured using the calibrated scoops provided with the material, ensuring an accurate powder-to-liquid ratio determination. The method of preparing the sample for testing and the tests performed are presented schematically in Figure 1.

Objective: Testing the ISO normative requirements and ion release from materials at pH = 4 and pH = 7 after 24 h and 30 days.

### 2.1. Setting Time

Sealers that are used to fill root canals in a tooth should have a suitable working time and setting so that they are convenient to use. This study followed the ISO 6876:2012 standard for root canal sealing materials [13]. The procedure involved mixing the powder and liquid components of each material and placing the mixture in a mold within a thermostatically controlled chamber maintained at 37 °C and 100% humidity. A Gilmore-type metric indenter (St-1 Lapka, Nova Paka, Czech Republic), which meets the normative requirements, was used to measure the setting time. The indenter, with a mass of 100.0 ± 0.5 g and a flat end diameter of 2.0 ± 0.1 mm, was lowered from a height of 5 mm on the material surface. The setting time was determined when the indenter no longer penetrated the material. Five samples were prepared for each type (according to the standard requirements), for a total of fifteen samples. ISO standard testing strictly defines the number of samples to be tested, limiting their number to 5 for each cement. If 4 out of 5 samples pass the test, then the entire material meets the standard. The setting time was recorded when the material mixing process started, and the final reading indicated no further indentation.

### 2.2. Consistency

The fluidity of the material is critical to ensure optimal flow into the narrow and complex spaces within the RC. Consistency was determined according to ISO 6876:2012, which specifies the standard procedure for measuring root canal sealing materials [13].

Five independent samples of each material were tested, totaling fifteen samples. The procedure involved placing 0.5 mL of freshly mixed material onto a clean, dry glass plate. Exactly 3 min after the start of mixing, a second glass plate was placed over the material. A reference weight was then added, bringing the total mass to 120 g, and the assembly was left for 10 min. After removing the load, the diameter of the circular spread was measured in two perpendicular directions using a precision caliper (Absolute, MITUTOYO, Kanagawa, Japan) with an accuracy of 0.1 mm. The results were recorded as the average of the two measurements for each sample.

### 2.3. Film Thickness

This test aimed to determine the thickness of the film formed by the material, a critical factor for its ability to seal and adapt to the root canal. ISO 6876:2012 standard was followed to ensure precise and consistent measurements [13].

Optically flat glass plates, circular in diameter (20 mm), with a minimum thickness of 5 mm and a contact area of 200 mm^2^, were thoroughly cleaned to remove contaminants. The initial height of two stacked glass cylinders was measured using a calibrated micrometer (ABS Digimatic Indicator ID-SX, MITUTOYO, Kanagawa, Japan) with 1 µm precision. The sealers, mixed according to the manufacturer’s instructions, were placed between the plates. After 3 min, a load of 150 N was applied to simulate clinical pressure. After 10 min, the height of the glass cylinders was remeasured with the material, and the difference between the final and initial heights indicated the thickness of the film. Five samples of each material were tested (according to standard requirements), yielding fifteen total samples.

### 2.4. Solubility

Another important property of materials for filling RC is their low solubility; for this purpose, the ISO 6876:2012 standard was used. Five samples for each material (fifteen in total), with fillings with mixed paste, as well as a metal mold diameter with a diameter of 20 mm and a thickness of 1 mm, were covered with optically flat glass plates to obtain a smooth surface [13]. After 24 h of curing, the molds were dismantled, and the samples were weighed on precision scales (Precisa, Dietikon, Switzerland) accurate to 0.0001 g, recording the initial mass as M1. The empty glass vessels (Pyerex Glass, Prague, Czech Republic) were also weighed and recorded as M2.

The samples were placed in glass vessels, and 50 mL of double distilled water was added. The vessels were sealed to prevent evaporation and contamination. After 24 h of immersion, the samples were removed, and the vessels were dried in a laboratory oven (TCF 50, Chromservis Cisterna di Latina, Italy) at 110 °C until the water evaporated. Once the glasses were cooled to room temperature in a desiccator, the final mass of the vessels (M3) was recorded.

The solubility was calculated using the following formula:

(1) $$S=\frac{M1-\left(M3-M2\right)}{M1}\times 100\%$$

where

M1 = Initial mass of the sample [g];
M2 = mass of the empty vessel [g];
M3 = mass of the vessel after drying [g].

### 2.5. Elemental Analysis

The safety of the materials will depend largely on the quantity of various substances released into the patient’s body. One of the methods that allows this to be determined in vitro is the analysis of releasing ions, tested by coupled plasma atomic emission spectrometry. The purpose of this study was to quantify the elemental content released from three types of sealers in solutions with pH levels of 4 and 7. Release tests were carried out after 24 h and 30 days to assess the dynamics of ion release over time. A total of 36 independent samples were prepared. Each material was tested in triplicate under both pH conditions to ensure reproducibility. Samples, 20 mm in diameter and 1 mm thick, were prepared following the procedure outlined in the Section 2.4 [14].

### 2.6. Ions Release Tests

The elemental release of the three sealers—Pulp Canal Sealer (Zn, Ag, and C), Kavitan Plus (Al, Na, Si, Ca, F, Sr, C, and P), and Adhesor (Zn, Al, Mg, and P)—is based on the compositions of materials available in the information leaflets and also in the SDS. The samples were stored at pH 4 (citrate) and pH 7 (Sigma-Aldrich, Poznan, Poland). Each sample was placed in a 50 mL polypropylene container containing the water, and the vessels were placed on an orbital shaker at 60 rpm. The samples were incubated at a constant temperature of 37 °C to simulate physiological conditions. The release was measured after 24 h and 30 days, and the samples were filtered and prepared for analysis after each time point. The experiments were carried out in triplicate, ensuring precision and repeatability (36 samples in total).

The extraction solutions were not re-invented during the experiment, which means that the concentration of released ions was cumulative over the testing period.

#### 2.6.1. Mineralization

The purpose of this test was to analyze the elements present in the tested materials, allowing quantification of those eluted during the test periods [15]. Three samples of each material (nine samples in total) were subjected to two-stage microwave-assisted mineralization in a closed, wet system using the START D microwave decomposition system (Milestone, Sorisole, Italy). Each sample weighed approximately 0.1 g and was placed in Teflon plates (Wuxi Rayflon Polymer Technology, Wuxi, China) for decomposition. 

First stage: Each sample received 2 mL of royal water (a mixture of 0.5 mL of nitric acid and 1.5 mL of hydrochloric acid) and 1 mL of hydrofluoric acid (all Suprapur purity acids, Merck, Darmstadt, Germany). Mineralization was carried out for 10 min at 100 °C with an oven power of 1000 W.

Second stage: After the first stage, 10 mL of boric acid (Merck, Darmstadt, Germany) was added to the premineralized materials. The process continued for 35 min, with temperatures ranging from 100 °C to 200 °C, and the oven was set to 1000 W. The mineralizates were then cooled and transferred to HDPE bottles, where they were diluted to a final weight of 50 g for further analysis.

#### 2.6.2. Analysis

The mineral composition—including Zn, Ag, Al, Na, Si, Ca, Sr, and P—was analyzed using inductively coupled plasma–atomic emission spectrometry (ICP-OES) with an iCAP 6500 Duo spectrometer (Thermo Fisher Scientific, Waltham, MA, USA). Analysis was carried out in both the extracts and the mineralized dental materials using validated methods that account for matrix effects. The tests were carried out at an accredited facility (AB 969) that specializes in multielement analysis.

The carbon content was determined separately using elemental analysis (Vario MACRO Cube, ELEMENTAR Analyzesysteme, Langenselbold, Germany) equipped with a thermal conductivity detector (TCD). Three samples from each reaction medium were used for the study (thirty-six in total).

#### 2.6.3. Analysis of Fluoride Concentration

The concentration of fluorides in the extract was determined by ion chromatography using a Dionex ICS 1100 ion chromatograph (Thermo Fisher Scientific, Waltham, MA, USA). The extract was injected directly into the chromatography column using a 0.2 m sterile syringe filter. For dental materials, an additional extraction was performed prior to analysis. Fluoride extraction: Add 50 mL of water chloride (HCl) to a 3 g dental gel (0.1 HCl) and then heat at 100 °C for an hour. The cooled solution was filtered into a 250 mL volume tube. The extracted samples were then filtered by sterile syringe filters and injected into a chromatographer for analysis [14].

Three samples of glass-ionomer cement were used for the test after 24 h and 30 days for pH = 4 and pH = 7 (12 samples in total).

##### Statistical Analysis

Statistical analysis was performed to assess the differences in the physical and chemical properties of the tested materials. One-way analysis of variance (ANOVA) was used to evaluate the variations between group means, with Tukey’s Honest Significant Difference (HSD) post hoc test applied for pairwise comparisons. A confidence level of *p* < 0.01 was established to ensure statistical significance.

All analyses were conducted using the online web statistical calculator tool (Online Web Statistical Calculators Navendu Vasavada 2016, New York, NY, USA) available at https://astatsa.com/OneWay_Anova_with_TukeyHSD/_get_data/ (accessed on 5 August 2024), ensuring the accuracy and reproducibility of the results.

Furthermore, multiple regression analysis evaluated the impact of the pH and time on ion release, which was performed using a multiple linear regression calculator (Mann–Whitney U test calculator [Internet], Statistics Kingdom 2017, London, UK) https://stats.blue/Stats_Suite/multiple_linear_regression_calculator.html (accessed on 5 August 2024).

## 3. Results

### 3.1. ISO Standard Requirements

The results of the tests based on ISO standards are presented in Table 2, which highlights the key distinctions between materials, particularly the longer set time and the higher fluidity of the Pulp Canal Sealer, while Adhesor and Kavitan showed faster set times.

The results indicate that the Pulp Canal Sealer exhibited a significantly longer setting time (62.6 ± 2.8 min, *p* < 0.01) compared to the Adhesor (6.49 ± 0.4 min) and Kavitan (4.36 ± 0.4 min).

In terms of solubility, the Pulp Canal Sealer has the lowest value (0.20 ± 0.05%), while Kavitan (0.33 ± 0.03%) was the highest, and Adhesor (0.07 ± 0.02%) had the lowest. This suggests better stability compared to those of the other materials.

The Pulp Canal Sealer also demonstrated the highest fluidity, with an average consistency of 36.8 ± 0.6 mm, which was statistically significantly higher (*p* < 0.01) compared to the other materials tested.

All three materials had film thicknesses of less than 25 microns, with no significant differences. This shows that all materials can form thin layers, which is important for achieving an accurate seal.

The differences in setting time, solubility, consistency, and film thickness highlight the unique properties of each sealer. Film thickness affects material adaptation to canal walls. ISO standards limit the thickness to 50 µm to prevent microleakage. The tested materials (0.22–0.26 µm) meet this requirement. The ZnO/eugenol-based materials show higher fluidity, facilitating tubule penetration, while GiCs offer strong dentin adhesion. The selection of the right material depends on clinical needs such as procedure time, sealing ability, durability, and bioactivity.

### 3.2. Ions Releasing

The results of the release of individual elements of the material for 24 h and 30 days stored at two different pH = 4 and pH = 7 are presented in Table 3 and Table 4 (Pulp Canal Sealer), Table 5 and Table 6 (Adhesor), and Table 7 and Table 8 for Kavitan.

Table 3 and Table 4 show that all trace elements in the material are released into the solution. The highest carbon content, derived from organic components such as eugenol and Canada balsam resin, remained stable regardless of the time or pH conditions, ranging from 208.7 ± 41.7 mg to 297.9 ± 59.6 mg, which represents approximately 0.7% of the total carbon content.

These data also show that the release of zinc, silver, and carbon from the Pulp Canal Sealer for 24 h and 30 days at pH levels of 4 and 7 was statistically significant, with *p* < 0.01. Zinc release increases significantly over time, with higher amounts observed after 30 days (1.3944 ± 0.2589 mg at pH 4 and 1.2923 ± 0.2784 mg at pH 7) compared to 24 h (0.1984 ± 0.0397 mg at pH 4 and 0.2576 ± 0.0515 mg at pH 7), suggesting a gradual breakdown of the zinc oxide-eugenol matrix. Sustained Zn^2+^ release enhances the antimicrobial effect of ZnO/eugenol-based sealers, as zinc disrupts bacterial membranes and inhibits metabolic enzymes. The prolonged ion release may help prevent secondary infections and improve long-term treatment success [5,7]). The slightly higher zinc release at pH 4 may result from faster ZnO dissolution under acidic conditions.

Silver release is minimal, consistently below the detection limit (<0.0050 mg), regardless of the pH or time, suggesting that silver remains predominantly bound within the material, potentially contributing to its antimicrobial effect while exhibiting negligible environmental release.

The release of aluminum is time-dependent but not influenced by the pH. After 30 days, the aluminum content is double that released after 24 h, which is statistically significant at *p* < 0.01, accounting for approximately 0.03% of the total aluminum concentration in the Adhesor sample. Physical and chemical processes control ion release. Phosphate and glass-ionomer materials degrade through hydrolysis and ion exchange, while ZnO/eugenol-based materials release ions via diffusion. Higher zinc release at an acidic pH suggests increased solubility, which may enhance the antimicrobial properties. Silicon and fluoride release at a neutral pH indicates slow glass matrix degradation, potentially aiding remineralization. Minimal silver release suggests its stable incorporation, reducing systemic exposure while maintaining antimicrobial potential.

Zinc release increases with time, reaching approximately 5% of the total zinc content after 24 h and 30% after 30 days in samples stored at pH 4. At a neutral pH, degradation is slower, with 3% released after 24 h and 7% after a month. But all of these values are statistically significant at *p* < 0.01

Magnesium release, ranging from 0.5% to 1.58% of the total content, is not affected by time and pH. The release is highest after 30 days at pH 4, reaching 1.5% of the total phosphorus content (statistically significant *p* < 0.01).

These data highlight the ion-release behavior of the Adhesor over time and under different pH conditions. Zinc release increases significantly at pH 4, where 30% is released after 30 days compared to 7% at a neutral pH. Zinc oxide dissolves more readily in acidic environments, where it forms soluble zinc cations, resulting in greater zinc release. At a neutral pH, the material is more stable, leading to slower zinc release.

In Table 8, it is visible that after 30 days at a pH 7—2.6% of the total aluminum content in the sample was released, which is statistically significant, *p* < 0.01. Sodium migration from the material follows a similar trend, with 12% of the total sodium after 30 days at pH 7. Silicon was detected in the solution (3.5% at pH 7 after 30 days), indicating degradation of the glass matrix and its role in promoting tissue remineralization. The calcium content was low (0.3%), and its release was below the detection limit.

The fluoride solubility in the solution is highest at pH 7 after 30 days, reaching 6.5% of the total fluoride content. (*p* < 0.01) Strontium release is also significant (*p* < 0.01) at 4.85% after 30 days at pH 7. Carbon release is minimal, between 0.17 and 0.22%, and is stable at different pH levels and time points.

These results highlight Kavitan’s ion-release behavior over time and under varying pH levels. At pH 7, aluminum, sodium, and silicon are released in increasing amounts, suggesting slow degradation of the glass matrix. GiCs degrade through controlled ion exchange. Sodium buffers the pH, silicon stabilizes collagen, and fluoride enhances remineralization. Aluminum contributes to mechanical strength but raises biocompatibility concerns in prolonged exposure.

### 3.3. Inter-Element Interactions

The following analysis is based on the data presented in Table 1, Table 2, Table 3, Table 4, Table 5, Table 6 and Table 7, which describe the composition, elemental release, and physicochemical properties of the materials. The data were further analyzed to explore inter-element interactions and the impact of the pH and time on the release of critical elements such as Zn, Ag, Al, and others. Using elemental release data from Table 3, Table 4, Table 5, Table 6 and Table 7, multiple regression models were constructed to assess the effects of the pH and time on zinc (Zn) and other key elements. The results are summarized below.

The correlation analysis for PC Sealer showed a strong positive correlation between the release of zinc (Zn) and carbon (C) (0.902), indicating that the release of these elements may be linked. Silver (Ag) did not show variability in this dataset, so its correlation with other elements could not be established.

For Adhesor, there were strong correlations between aluminum (Al) and magnesium (Mg) (0.985) and between zinc (Zn) and phosphorus (P) (0.979). These strong correlations suggest that the chemical release processes of these elements are closely interconnected (Table 9).

### 3.4. Regression Analysis

The regression analysis for PC Sealer demonstrated that the time factor has a significant positive effect on zinc (Zn) release, with a regression coefficient of 0.186. The pH factor had a minimal and negative influence on Zn release, with a coefficient of −0.007. The primary determinant of the release of Zn from PC Sealer is the duration of exposure, with higher release rates observed over longer periods. The effect of the pH is minimal but slightly negative, suggesting that lower pH conditions may slightly reduce the release of Zn.

The pH had a stronger negative effect on zinc (Zn) release, with a regression coefficient of −0.138, while time had a positive effect, with a coefficient of 0.107. The release of Zn in the Adhesor is more sensitive to changes in the pH, with lower pH conditions facilitating greater Zn release. Over time, Zn release increases, but the effect is less pronounced than in PC Sealer.

**Figure 2:**
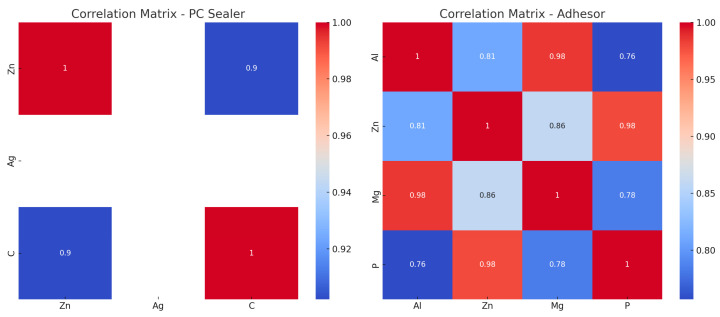
Heatmap illustrating inter-element correlations for PC Sealer and Adhesor.

The heatmap below illustrates the correlation matrices for PC Sealer and Adhesor, highlighting the relationships between the elements (Figure 2).

The correlation and regression analyses suggest distinct behavior for PC Sealer and Adhesor in terms of element release. For PC Sealer, zinc release is largely time-dependent, with minimal impact from the pH. In contrast, the Adhesor shows a stronger sensitivity to pH, especially under acidic conditions, where zinc release is more pronounced.

## 4. Discussion

The materials used in these studies belong to various traditional root canal-filling materials that have been used in dentistry for more than 60 years. Each of them belongs to a different group of materials having specific properties. The initial hypothesis was partially confirmed since all the materials tested met the minimum standards for filling materials for the root canal. However, significant differences in ion release were observed, influenced by the pH of the environment (pH = 4 and pH = 7).

The materials used in the tests are characterized by different reaction rates. Glass ionomer and phosphate cements have a very fast setting time. After their hardening, hard material is obtained, in which incompletely reacted powder particles (zinc oxide–phosphate cements) or glass, in the case of glass-ionomer cements, are placed in a matrix of acid salts (phosphates or polyacrylic acid). The large molecule polyacrylic acid impairs the solubility of the cement compared to the phosphoric acid contained in phosphate cements. This leads to a situation where zinc cations are more easily washed out from the salt matrix or even from the unreacted filler particles themselves in an acidic environment. Aluminum cations form a spatial crystal lattice, which reduces their solubility. In the case of cement based on zinc oxide and eugenol, after the reaction, a complex of these two components is formed, which is degraded under the influence of water, which causes a greater release of zinc ions into the reaction environment.

The variations are linked to the chemical composition and stability of the materials under different pH conditions. For example, the higher release of Zn^2+^ ions from ZnO-based materials at an acidic pH can be attributed to the breakdown of the ZnO matrix in the presence of excess H^+^ ions, which facilitates the release into the solution. This effect becomes more pronounced after prolonged exposure (30 days), with up to 30% of the total Zn released from phosphate sealants under acidic conditions [16,17].

Clinically, these differences in ion release are significant. The hypothesis proposed that the ion-release patterns could influence the clinical applications of each material. Zinc oxide-eugenol sealers were expected to be particularly suitable for cases with active infections because of their higher ion release in acidic environments, which increased their antimicrobial properties. On the contrary, glass-ionomer sealers, with their higher release of fluoride and strontium ions at a neutral pH, were hypothesized to be more effective in promoting remineralization and tissue repair.

Recent studies have explored the incorporation of nanoparticles into endodontic sealants to enhance antimicrobial properties. For example, the addition of zinc oxide nanoparticles to traditional cements has significantly improved antimicrobial efficacy while maintaining mechanical integrity [5]. Such innovations indicate the ongoing evolution of root canal materials and nanotechnology that offer improved outcomes in infection control. Zinc ions are particularly known for their antibacterial effects, and their increased release in acidic environments may help with root canal infections. Although released in smaller amounts, silver ions may also contribute to these antimicrobial effects [9,10].

Other studies have emphasized the advantages of bioceramic sealers over traditional zinc oxide-based materials due to their biocompatibility and ability to promote healing in periapical tissues. ZnO-based sealers exhibit superior physical properties, such as better flow and lower solubility, which contribute to greater durability in clinical use [11,12,18]. This shift away from traditional zinc oxide-based materials offers both antimicrobial efficacy and support for periapical healing [19].

Bioceramic sealants, including calcium silicate-based materials, exhibit superior ion release, particularly calcium and hydroxide ions, which create an alkaline environment conducive to tissue regeneration and reduced inflammation. The material with strontium-doped nanohydroxyapatite significantly improved remineralization, further demonstrating the potential of advanced ceramic technology in modern dentistry [20]. The materials, with controlled ion-release profiles, balance antimicrobial properties with bioactivity, promoting healing while preventing reinfection in root canal treatments.

The release of fluoride from glass-ionomer sealers (GiCs) at a neutral pH prevents secondary caries by starting the remineralization process of dentin and enamel. Fluoride promotes the formation of fluorapatite, which is more resistant to acid [21,22]. Glass ionomer cements provide sustained fluoride release, enhancing enamel and dentin resistance to demineralization. The incorporation of fluoride into hydroxyapatite reduces solubility in acidic conditions, thereby benefiting patients at high risk of caries. In the literature, it is possible to find that fluoride release depends on the material formulation, with some variations releasing higher amounts of fluoride, offering superior protection in cases of recurrent caries or erosion. These findings are consistent with previous studies showing that fluoride-rich materials provide sustained protection against demineralization [23].

The release of silicon (Si) and strontium (Sr) ions from GiCs is also pH-dependent, with a greater release at a neutral pH (pH = 7). This may be due to the gradual dissolution of the silica network and the solubility of strontium salts in neutral environments. Strontium supports remineralization, similar to fluoride, and aids bone regeneration. The increased release of these ions in neutral environments highlights the bioactivity of GiCs in promoting tissue repair, especially under non-acidic conditions [24,25].

Variations in ion release reflect the intrinsic properties of the materials and their susceptibility to environmental pH. Clinically, these differences can guide material selection based on specific treatment needs. Zinc oxide-based materials may be more effective for infection control, while glass-ionomer sealers may be preferable in cases where remineralization and tissue repair are priorities. The physical properties of the materials, such as film thickness, setting time, and solubility, are aligned with their clinical uses. The extended setting time of ZnO/eugenol-based sealants (62.6 ± 2.8 min) provides a prolonged working phase but increases the risk of contamination. On the contrary, phosphate cements set quickly (4.36–6.49 min), improving stability but limiting adaptability to dentin.

Zinc oxide-eugenol-based materials, such as Pulp Canal Sealer, are known to have longer setting times, which can be beneficial when more time is needed to position the material in the root canal. Longer setting times allow precise placement but may slow the workflow and require careful handling to prevent displacement before polymerization. In contrast, bioceramic sealers set without shrinkage, reducing microleakage risk over time [2,14]. Faster-setting adhesives and Kavitan may be preferable in procedures that require faster completion, although they may pose a higher risk of errors if not properly managed.

Another dependency is the thickness of the film. For example, Pulp Canal Sealer demonstrated a film thickness of 0.22 ± 0.02 μm, a setting time of 60 min, a consistency of 36.8 ± 0.6 mm, and a solubility of 0.20 ± 0.05%. Although slightly different from the values reported in the literature, these measurements remain within the acceptable range for root canal sealing materials [26]. The thickness of the Kavitan film of 23 μm is consistent with previous studies and meets the ISO standard [27,28]. The phosphate sealants had a film thickness of 0.26 μm [28]. The thinner film thickness of 0.23 μm makes it ideal for applications that require a minimal material layer thickness. Solubility is a critical factor for long-term performance. Solubility (0.33%) falls within the reported range of 0.3–1.3%, which supports its long-term stability. The adhesive showed even lower solubility (0.07%), which is consistent with other tests [29,30].

The time to establish the filling materials of the root canal-filling materials must meet specific criteria to ensure proper mixing and application before hardening. The ZnO/phosphate sealer should set in 4–8 min [31,32]. Kavitan, a glass-ionomer cement (GiC), had a set time of 4 min and 30 s, which is within the appropriate range for this type of material [33]. This provides sufficient working time without the material hardening too quickly, allowing for precise placement in the root canal.

Given the prolonged contact of these sealers with biological tissues, any released ions are non-toxic and preferably bioactive. Newer bioactive material formulations aim to optimize ion release to balance biocompatibility with antimicrobial activity. Research has shown that modern glass-ionomer and bioceramic materials not only release beneficial ions, such as calcium and fluoride, but also maintain low cytotoxicity, which is significant for long-term success in endodontic therapy. The bioactivity of these sealers could promote remineralization by providing essential ions such as calcium, fluoride, phosphate, and strontium [16,33,34].

Zinc oxide-eugenol sealers release these elements over time. Gradual Zn^2+^ ion release provides sustained antibacterial action but may cause cytotoxicity if the material extends beyond the apex. ZnO/eugenol-based materials show antimicrobial effects as a result of zinc ions and eugenol, which disrupt bacterial membranes. However, their cytotoxicity risks must be considered in overfilled cases. In contrast, GiCs release fluoride, offering long-term protection against secondary caries. Careful handling is required, particularly for patients sensitive to zinc. Eugenol is released rapidly within the first 24 h, as evidenced by the carbon content in the tested samples (243.4 ± 48.7 mg after 24 h and 284.5 ± 56.9 mg after 30 days). This initial burst of unreacted eugenol is followed by a slower release as the zinc oxide-eugenol complex gradually hydrolyzes [35,36]. Zinc ions are also released steadily, increasing even after 30 days, regardless of the environmental pH. In the case of other materials, zinc oxide phosphate sealer releases zinc, magnesium, and phosphate ions in acidic environments (pH = 4). Under such conditions, up to 30% of the total zinc content can be released. This occurs because of the incomplete reaction of ZnO particles that remain surrounded by the products of the zinc oxide-phosphoric acid reaction. At a low pH, these residual ZnO particles react more readily, releasing zinc cations into the solution [22]. The explanation of this phenomenon can be explained by the formation of soluble salts such as Zn(HPO_4_) and Mg (HPO_4_), which leads to increased ion release [17].

For glass-ionomer material, the release of ions such as sodium and silicon is consistent with their chemical composition. Recent studies have also explored the role of strontium ions in enhancing the bioactivity of glass ionomers. The literature describes that strontium-doped glass ionomers not only promote remineralization but also improve mechanical properties, such as increased fracture toughness, compared to traditional formulations [22]. Advances in ionomer formulations can enhance the therapeutic and structural properties of materials. At a neutral pH (pH = 7), sodium ions are steadily released, reaching concentrations of 1480 ppm after 30 days [33,34]. Additionally, silicon and aluminum ions are released in greater quantities in neutral environments, further enhancing the bioactivity of the material. The ions contribute to tissue repair, making glass ionomers particularly beneficial in cases requiring tissue regeneration and remineralization. Calcium silicate-based sealers release bioactive ions that promote mineralization. The alkaline pH from Ca^2+^ and OH^−^ ions supports hydroxyapatite formation but may induce initial cytotoxicity. Their prolonged setting time (>24 h) limits the immediate mechanical stability compared to conventional sealers. The low calcium (Ca) release observed during the tests raises questions about the behavior of calcium-containing components in materials. The minimal release of calcium, regardless of the pH, may be due to the formation of stable, undissolved calcium compounds, such as orthophosphate or monofluorophosphate, on the surface of the material. These compounds could create a protective layer that limits further calcium dissolution [33].

Bioceramic sealants are known for their controlled release of calcium ions that contribute to their biocompatibility and the ability to promote mineralized tissue formation and the creation of a bioactive environment while showing reduced cytotoxicity compared to traditional zinc oxide-based sealants [19]. Their ability to facilitate the formation of hydroxyapatite at the material–tissue interface supports long-term healing and reduces post-treatment inflammation, offering a significant clinical advantage in endodontic therapies where long-term biocompatibility and sustained healing are achieved.

The relatively high release of silicon (Si) at a neutral pH (pH 7) in a glass-ionomer sealer can be attributed to the high solubility of silicon compounds in water. Silicon is often present as sodium silicate compounds (Na_2_O·nSiO_3_, where *n* = 1–3.35), which exist in solution as dimers or trimers. The highly soluble forms readily dissolve in water, explaining the elevated levels of silicon observed in extracted solutions [33,35].

The release of aluminum (Al) in neutral environments can be explained by the dissolution of aluminum-containing salts, such as silicates and alumino-silicates. The salts form dissolved oligomers, increasing the concentration of aluminum ions in solution due to the breakdown of these complex salts under neutral conditions [24,36].

Another factor to consider is the high solubility of glass ionomers (GiCs), such as Kavitan, when hand-mixed. Hand mixing may lead to incomplete reactions between the glass particles and acrylic acid, the primary liquid component. As a result, unreacted glass particles may dissolve more rapidly in solution, leading to a higher level of ion release due to incomplete reactions during manual mixing, which could explain the increase in solubility and faster dissolution rates observed in this study [24].

Fluoride ion (F^−^) release is one of the key benefits of glass-ionomer-based materials because fluoride has well-established bacteriostatic properties and improves the hardness of both enamel and dentin. Additionally, recent advances in fluoride-releasing dental materials have focused on optimizing fluoride-release kinetics for long-term sustained protection. The modified glass ionomer can release significantly higher levels of fluoride ions over extended periods, offering enhanced protection against caries formation in high-risk patients. This represents a significant advance in preventive dentistry, providing clinicians with materials capable of long-term resistance to caries [22]. In the case of the Kavitan material, fluoride ions were released more abundantly at a neutral pH (pH 7) [17]. The mechanism behind this release can be attributed to ion exchange processes. During this process, fluoride ions are exchanged with hydroxide ions derived from the storage solution, allowing fluoride to be released without causing a significant loss of structure in the material [37]. The amount of fluoride released by different glass-ionomer materials can vary depending on their composition. In this study, the fluoride release ranged between 2 and 3.3 mg/L after 30 days, while the literature reported much higher fluoride release values of up to 20 mg/L for Fuji II cement [22,23]. The differences highlight the variability in fluoride release among different formulations of glass ionomer, indicating that the specific composition of each material plays an important role in its clinical performance.

Despite the valuable information gained, this study has certain limitations. The ion release was analyzed for a limited group of commercially available materials, and the composition was based on information provided by the manufacturers (instruction manuals and safety data sheets). Materials contain additional elements, such as heavy metals, which were not measured in this study but could be released over time [24]. Future studies should investigate the presence and release of such elements to ensure comprehensive safety assessments of these materials. Research on alternative bioactive fillers, such as hydroxyapatite and bioactive glass, may reduce the dependence on aluminum and zinc. Nanoparticle-modified materials show promise for improved biocompatibility and antimicrobial efficacy. Furthermore, recent research has emphasized the need for long-term in vivo studies to evaluate the potential cumulative effects of trace elements, such as aluminum and heavy metals, released from dental materials that have long contact with the patient. This study was performed for traditional materials; modern materials based on bioceramics can release ions in other quantities over time and at pH. Therefore, in our future work, we will try to analyze the behavior of other groups of materials intended for filling the root canal. A publication raised concerns about the release of trace elements from glass-ionomer materials, highlighting the importance of continuous monitoring to ensure their biocompatibility and safety over time [24,38,39]. 

These findings lay the foundation for future research to improve biocompatibility and optimize the ion release of root canal-filling materials. Future studies could focus on refining its chemical composition to enhance mechanical properties and therapeutic effects while also examining a wider range of materials under varying clinical conditions, such as temperature and microbial activity, to obtain better in vivo insights. Differences in setting time, solubility, and ion release among the tested materials provide clinicians with a guide to select the most appropriate material based on the patient’s needs and desired therapeutic results.

## 5. Conclusions

This study examines how material composition influences ion release under different pH conditions over time. The interaction between material chemistry and environmental factors affects bioactivity, stability, and antimicrobial effects. Comparative analysis of zinc oxide/eugenol, phosphate, and glass-ionomer materials provides insight into their long-term performance. These findings may aid in optimizing formulations and clinical applications.

Higher zinc release from ZnO/eugenol-based materials under acidic conditions suggests their suitability for infected root canals, where antimicrobial effects are needed. However, prolonged zinc elution may cause tissue irritation, requiring case-by-case evaluation. Increased silicon and fluoride release from glass-ionomer materials at a neutral pH can support dentin remineralization, making them useful in cases requiring bioactive effects. Phosphate-based materials show controlled ion release, supporting their use in temporary applications rather than permanent sealers. Selecting the right material depends on the infection status, pH variations, and long-term stability requirements.

All tested materials met the regulatory requirements for root canal filling. The silver in the ZnO/Eugenol sealer remained below the detection threshold, suggesting a minimal elution and a negligible risk of systemic exposure. Zinc ion release from ZnO/eugenol and ZnO/phosphate sealers increased significantly under acidic conditions. This pH-dependent release emphasizes the need for material selection based on the clinical environment, especially in acidic conditions associated with infections. At a neutral pH, the glass-ionomer material released substantial amounts of silicon and strontium, potentially enhancing enamel and dentin remineralization. This study highlights the importance of time and pH in influencing ion release from PC Sealer and Adhesor.

## Figures and Tables

**Table 1:** Materials used for testing.

Material Name and Producer	Type of Sealers	Raw Composition Based on SDS	Mixing Ratio (Powder/Liquid) [g]
Pulp Canal Sealer (Kerr, Scafati, Italy) Batch: 765,439	Zinc oxide/eugenol	Liquid: eugenol, Canada balsam Powder: zinc oxide, silver, thymol iodide	0.17/0.03 [g] *
Kavitan (SpofaDental, Jicin, Czech Republic) Batch: 9,492,008	Glass ionomer cement	Liquid: polyacrylic acid, tartaric acid, Water powder: glass	1.5/0.5 [g] *
Adhesor (SpofaDental, Jicin, Czech Republic) Batch: 7,984,355	Zinc oxide phosphate cement	Liquid: phosphoric acid, aluminum hydroxide, water Powder: zinc oxide, magnesium oxide, aluminum hydroxide	2.0/0.5 [g] *

* Mixing proportions were determined by weighing the amount of material recommended by the manufacturer. Liquids were measured in drops (bottles with droppers), and powders were measured using the spoons provided in the respective sets.

**Table 2:** Results of the setting time, consistency, film thickness, and solubility of the tested materials, Mean ± SD.

	Setting Time [min]	Consistency [mm]	Film Thickness [µm]	Solubility [%]
Pulp Canal Sealer	62.6 ± 2.8 *	36.8 ± 0.6 *	0.22 ± 0.02	0.20 ± 0.05 *
Adhesor	6.49 ± 0.4	32.7 ± 0.5 *	0.23 ± 0.01	0.07 ± 0.02
Kavitan	4.36 ± 0.4	34.1 ± 0.5 *	0.22 ± 0.02	0.33 ± 0.03

Note: Statistically significant values are indicated by * for *p* < 0.01.

**Table 3:** Percentage of elements in pulp canal sealer material (PC Sealer) subjected to the mineralization process sample 1 g.

PC Sealer	Concentration ± SD [%]
Zinc (Zn)	18.47 ± 3.69
Silver (Ag)	0.038 ± 0.006
Carbon (C)	18.90 ± 3.78

**Table 4:** Content of zinc, silver, and carbon from PC Sealer material per 1 g sample.

pH	Time	Zn ± SD [mg]	Ag ± SD [mg]	C ± SD [mg]
4	24 h	0.1984 ± 0.0397 *	<0.0050 ± 0.001	226.8 ± 45.3
	30 days	1.3944 ± 0.2589 *	<0.0050 ± 0.001	273.2 ± 54.6
7	24 h	0.2576 ± 0.0515 *	<0.0050 ± 0.001	245.4 ± 49.1
	30 days	1.2923 ± 0.2784 *	<0.0050 ± 0.001	258.4 ± 51.7

Note: Statistically significant values are indicated by * for *p* < 0.01.

**Table 5:** Percentage of elements in the Adhesor material subjected to the mineralization process sample 1 g.

Adhesor	Concentration ± SD [%]
Aluminum (Al)	1.25 ± 0.25
Zinc (Zn)	19.43 ± 2.91
Magnesium (Mg)	3.50 ± 0.70
Phosphorous (P)	5.81 ± 1.16

**Table 6:** Elements released from Adhesor at different pHs and times (1 g sample).

pH	Time	Al ± SD [mg]	Zn ± SD [mg]	Mg ± SD [mg]	P ± SD [mg]
4	24 h	0.0059 ± 0.0012 *	0.0891 ± 0.013 *	0.0868 ± 0.0173 *	0.0270 ± 0.0054 *
	30 days	0.0228 ± 0.0046 *	1.1140 ± 0.167 *	0.3175 ± 0.0635 *	0.1209 ± 0.0242 *
7	24 h	0.0077 ± 0.0015 *	0.0610 ± 0.0091	0.0656 ± 0.0131 *	0.0433 ± 0.0087
	30 days	0.0206 ± 0.0041 *	0.3166 ± 0.0475	0.2609 ± 0.0522 *	0.0471 ± 0.0094

Note: Statistically significant values are indicated by * for *p* < 0.01.

**Table 7:** Percentage of elements in the Kavitan material subjected to the mineralization process (Sample 1 g).

Kavitan	Concentration ± SD [%]
Aluminum (Al)	10.45 ± 2.09
Sodium (Na)	2.38 ± 0.36
Silicon (Si)	9.54 ± 1.91
Calcium (Ca)	3.1 ± 0.06
Fluorine (F)	5.55 ± 0.25
Strontium (Sr)	11.86 ± 2.37
Carbon (C)	4.62 ± 0.69

**Table 8:** Elements extracted from 1 g of Kavitan sample concentration of elements extracted at pH 4 and pH 7 over time.

pH	Time	Al ± SD [mg]	Na ± SD [mg]	Si ± SD [mg]	Ca ± SD [mg]	F ± SD [mg]	Sr ± SD [mg]	C ± SD [mg]
4	24 h	0.0524 ± 0.0105	0.477 ± 0.0715 *	0.058 ± 0.0116 *	<0.0050 ± 0.001	0.8120 ± 0.1218 *	0.009 ± 0.0018	0.113 ± 0.0169
	30 days	0.100 ± 0.020	2.150 ± 0.323 *	0.357 ± 0.071 *	<0.0050 ± 0.001	2.102 ± 0.315 *	0.010 ± 0.002	0.110 ± 0.016
7	24 h	0.0536 ± 0.011 *	0.4340 ± 0.0651 *	0.041 ± 0.0081 *	<0.0050 ± 0.001	0.7215 ± 0.1082 *	0.002 ± 0.0004 *	0.1486 ± 0.0223
	30 days	0.7207 ± 0.1441 *	15.01 ± 2.2515 *	1.0296 ± 0.2059 *	<0.0050 ± 0.001	3.3443 ± 0.5017 *	1.181 ± 0.2362 *	0.131 ± 0.0197

Note: Statistically significant values are indicated by * for *p* < 0.01.

**Table 9:** Correlation matrices for PC Sealer and Adhesor and multiple regression coefficients for zinc (Zn) release as a function of pH and time for both materials.

(a) Correlation matrix for PC Sealer elements (Zn, Ag, and C).				
**Element**	**Zn**		**Ag**	**C**
Zn	1.000		NaN	0.902
Ag	NaN		NaN	NaN
C	0.902		NaN	1.000
(b) Correlation matrix for Adhesor elements (Al, Zn, Mg, and P).				
**Element**	**Al**	**Zn**	**Mg**	**P**
Al	1.000	0.810	0.985	0.757
Zn	0.810	1.000	0.859	0.979
Mg	0.985	0.859	1.000	0.783
P	0.757	0.979	0.783	1.000
(c) Multiple regression coefficients for PC Sealer (Zn release as a dependent variable).				
**Variable**		**Coefficient (Zn)**		
pH		−0.007		
Time		0.186		
(d) Multiple regression coefficients for the adhesion Adhesor (Zn release as a dependent variable).				
**Variable**		**Coefficient (Zn)**		
pH		−0.138		
Time		0.107		

## Data Availability

The original contributions presented in this study are included in the article. Further inquiries can be directed to the corresponding author.
